# Clinical features and immunotherapy outcomes in antibody-negative autoimmune encephalitis: a retrospective case–control study

**DOI:** 10.3389/fneur.2024.1464165

**Published:** 2024-09-12

**Authors:** Weiwei Gao, Jingjing She, Lihong Su, Shouyue Jin, Qingwei Yang, Xingyu Chen, Renjing Zhu

**Affiliations:** Department of Neurology, School of Medicine, Zhongshan Hospital of Xiamen University, Xiamen University, Xiamen, China

**Keywords:** autoimmune encephalitis, immunotherapy response, clinical features, antibody-negative, intrathecal IgG synthesis, albumin quotient

## Abstract

**Objective:**

This study aimed to compare clinical features, laboratory findings, and immunotherapy responses between antibody-positive and antibody-negative Autoimmune encephalitis (AE) patients.

**Methods:**

A retrospective analysis of clinical data from 60 AE patients (33 antibody-positive, 27 antibody-negative) diagnosed at Zhongshan Hospital of Xiamen University between January 1, 2016, and March 1, 2024 was conducted. Disease severity and treatment response were assessed using the modified Rankin Scale (mRS) and the Clinical Assessment Scale for Autoimmune Encephalitis (CASE).

**Results:**

Antibody-positive AE patients more frequently presented with multiple symptoms (≥4 symptoms: 39.4% vs. 14.8%, *p* = 0.036). They demonstrated significantly elevated serum IgG concentrations (*p* = 0.010) and cerebrospinal fluid (CSF) leukocyte counts (*p* = 0.014). Conversely, antibody-negative AE patients presented with higher CSF total protein levels (*p* = 0.025) and albumin quotients (*p* = 0.018), indicative of more severe blood–brain barrier disruption. Antibody-positive AE patients more frequently received combination first-line immunotherapy (75.8% vs. 48.1%, *p* = 0.027) and exhibited superior treatment outcomes (90.9% vs. 70%, *p* = 0.022). Among critically ill patients (peak mRS score: 4–5), improvement in CASE scores was markedly greater in the antibody-positive cohort (median: 4.50 vs. 1.00, *p* = 0.024).

**Conclusion:**

Antibody-positive AE patients manifested a more diverse symptom spectrum, elevated serum IgG concentrations and CSF leukocyte counts, and superior responses to immunotherapy. In contrast, antibody-negative AE patients demonstrated more severe blood–brain barrier dysfunction, as evidenced by higher CSF total protein concentrations and albumin quotients.

## 1 Introduction

Autoimmune encephalitis (AE) encompasses a spectrum of inflammatory central nervous system disorders mediated by autoimmune responses. AE is characterized by acute or subacute onset and presents with a diverse and complex array of clinical manifestations. Patients may concurrently experience cognitive impairment, psychiatric and behavioral abnormalities, seizures, speech and sleep disorders, and movement disturbances (1). Epidemiological studies estimate that the annual incidence of AE is 0.8 cases per 100,000 people, with a prevalence of 13.7 cases per 100,000 people, exhibiting a year-on-year upward trend (2). The ramifications of AE are multifaceted and extensive. Patients often endure protracted neurological dysfunction, compounded by a propensity for relapse and prolonged recovery periods. Furthermore, the substantial costs associated with AE management impose a significant financial burden on patients and their families. Collectively, these factors exert considerable strain on individuals, households, and healthcare systems, elevating AE to a formidable public health challenge (3).

Traditionally, AE diagnosis relies on the detection of neuronal autoantibodies in serum and cerebrospinal fluid (CSF). However, a substantial subset of AE patients lack detectable neuronal surface or synaptic protein autoantibodies, a phenotype designated as antibody-negative AE. Previous studies have reported that these patients constitute 42.1–50.7% of all AE patients (4–6). Antibody-negative AE patients frequently present with nonspecific clinical manifestations, unremarkable CSF profiles, and atypical neuroimaging features. This constellation of findings poses diagnostic challenges, potentially leading to treatment delays and inappropriate therapeutic interventions, which may adversely impact patient outcomes.

The underlying pathophysiological mechanisms of antibody-negative AE remain elusive. Several hypotheses have been proposed: autoantibody titers below current detection thresholds; the presence of novel yet-to-be-identified antibodies targeting neuronal surface or synaptic proteins; and atypical immune mechanisms, such as non-antibody-mediated cytotoxic T-cell responses (1, 7, 8). These hypotheses not only challenge our conventional understanding of AE pathogenesis but also necessitate a critical re-evaluation of the applicability and efficacy of current diagnostic criteria and therapeutic strategies for AE.

Graus et al. proposed the first clinical diagnostic criteria for antibody-negative AE (1), a landmark development that facilitated identification and research of this subtype, garnering significant attention within the medical community. Despite this progress, systematic comparative studies of clinical features and immunotherapy responses between antibody-negative and antibody-positive AE patients remain limited.

This study aimed to contribute to the growing body of knowledge on AE by conducting a comprehensive comparison of antibody-negative and antibody-positive cases. While previous studies have explored various aspects of AE, our research seeks to extend this understanding by systematically analyzing the similarities and differences in clinical manifestations, CSF biomarkers, and immunotherapy responses between these two groups within our specific geographic and demographic context. This regional perspective may offer unique insights into potential variations in disease presentation and management across different populations. Our objective is to refine and expand upon existing diagnostic and treatment strategies for antibody-negative AE, potentially enhancing early recognition and management of these challenging cases. Furthermore, this study contributes a substantial dataset to the existing literature, which may serve as a valuable reference for future meta-analyses and systematic reviews.

## 2 Methods

### 2.1 Study design and participants

A single-center retrospective study was conducted at Zhongshan Hospital of Xiamen University, enrolling 152 consecutive patients with suspected AE between January 1, 2016, and March 1, 2024. Antibody-positive AE was diagnosed based on the detection of specific neuronal surface or synaptic protein antibodies in CSF and serum samples. Antibody-negative AE was diagnosed according to the clinical criteria proposed by Graus et al. (1). Two experienced neurologists independently evaluated all potential participants, with disagreements resolved through discussion or consultation with a third expert.

We initially excluded 19 patients who did not undergo antibody testing. The remaining 131 patients who completed antibody testing underwent further evaluation. Among these, we excluded 43 antibody-negative patients subsequently diagnosed with infectious encephalitis or other conditions, 10 patients who did not meet the diagnostic criteria for possible or probable AE, and 20 patients whose clinical data were incomplete. After this rigorous selection process, our final study cohort comprised 60 patients: 33 with antibody-positive AE and 27 with antibody-negative AE.

Inclusion criteria for antibody-negative AE were: (1) age ≥ 14 years; (2) acute or subacute onset (symptom progression <3 months), manifested as working memory deficit, altered mental status, psychiatric symptoms, seizures, language dysfunction, movement disorders, gait instability, or limb weakness; and (3) at least one of the following: unexplained seizures, CSF leukocytosis (>5 cells/mm^3^), CSF-specific oligoclonal bands or elevated IgG index (>0.85), increased CSF protein levels, or magnetic resonance imaging (MRI) findings suggestive of autoimmune encephalitis.

Exclusion criteria were: (1): laboratory-confirmed infectious encephalitis (e.g., caused by herpesviruses, enteroviruses, or other infectious pathogens); (2) toxic/metabolic encephalopathy, brain tumor, or vitamin deficiency diagnosed before AE onset; (3) primary psychiatric disorders; (4) other specific immune-mediated encephalitides (such as Hashimoto’s encephalopathy and Bickerstaff brainstem encephalitis); and (5) incomplete clinical data.

### 2.2 Data collection

A comprehensive dataset was compiled using a predesigned structured electronic data collection form, and information was extracted from the hospital’s electronic medical records system. The collected data included demographic characteristics (sex and age), clinical features (medical history, prodromal symptoms, initial presenting symptoms, and specific clinical manifestations), neuroimaging and electrophysiological findings (MRI and EEG results), and treatment-related information (types of first-line immunotherapy administered, time interval between symptom onset and initiation of immunotherapy, and duration of hospital stay). The laboratory parameters obtained within 24 h of admission included hematological indices (complete blood count, serum protein concentration, C-reactive protein, and serum protein electrophoresis) and CSF analysis (pressure, cytology, biochemical markers, and immunological parameters). Additionally, we calculated several composite indicators, including inflammatory markers (neutrophil-to-lymphocyte ratio [NLR] and monocyte-to-lymphocyte ratio [MLR]) and CSF-specific parameters (albumin quotient [QAlb], IgG index, intrathecal IgG synthesis rate, and 24-h intrathecal IgG synthesis rate). The methods for calculating these derived parameters are detailed in Table 1.

**Table 1:** Formulas for calculated CSF parameters.

Parameter	Formula
Neutrophil-to-Lymphocyte Ratio (NLR)	Neutrophil count/Lymphocyte count
Monocyte-to-Lymphocyte Ratio (MLR)	Monocyte count/Lymphocyte count
Albumin Index (QAlb)	(Alb_CSF_/Alb_serum_) × 1,000
IgG Index	(IgG_CSF_/ IgG_serum_)/(Alb_CSF_/Alb_serum_)
IgG Local Synthesis (IgG (loc))	{Q_IgG_ – 0.8 × [Q_Alb_^2^ + (15 × 10^−6^)]^0.5^ + 1.8 × 10^−3^} × IgG_serum_
IgG Synthesis Rate (IgG SR)	[(IgG_CSF_ − IgG_serum_/369) − (Alb_CSF_ − Alb_serum_/230)× IgG_serum_/(Alb_serum_ × 0.43)] × 5

Unit standardization - Alb_CSF_ and IgG_CSF_ (mg/L) divided by 10 to convert to mg/dL; IgG_serum_ and Alb_serum_(g/L) multiplied by 100 to convert to mg/dL. All parameters analyzed in mg/dL. The numbers 369 and 230 are the averages in normal serum: CSF ratios for IgG and albumin, respectively. The number 0.43 is the molecular weight ratio of albumin. Five (5) is the daily CSF production expressed in dL.

### 2.3 Antibody testing

A comprehensive panel of AE-related antibodies was tested in serum and CSF samples from all enrolled patients. The panel included antibodies against neuronal cell surface antigens (NMDAR, LGI1, CASPR2, AMPAR, GABAAR, GABABR, DPPX, GlyR, and IgLON5) and intracellular antigens (GAD65, SOX1, Hu, Yo [PCA-1], Ri [ANNA-2], Ma1, Ma2, CV2, and amphiphysin). All antibody tests were performed at Guangzhou KingMed Diagnostics Laboratory using a multistep approach. Initially, rat brain sections were subjected to immunohistochemistry. Subsequently, neuronal cell surface antibodies were detected using indirect immunofluorescence (IIFT) on commercially available HEK293 cells expressing the relevant antigens. Intracellular antibodies were identified by Western blotting. To ensure reliability of the results, each sample was analyzed in duplicate. Antibody negativity was defined as the absence of all tested autoantibodies in both the serum and CSF. To eliminate potential treatment effects on antibody status, all samples were collected and analyzed upon admission prior to the initiation of immunotherapy.

### 2.4 Classification of disease severity and treatment response

Patient condition was comprehensively evaluated using the modified Rankin Scale (mRS) and the Clinical Assessment Scale for Autoimmune Encephalitis (CASE). Assessments were conducted at three critical time points: admission, peak disease severity, and discharge. The mRS primarily assesses overall functional status and degree of disability, with a focus on motor function. The CASE scale encompasses nine neurological domains: seizures, cognition, behavior, consciousness, language, movement/muscle tone, balance/coordination, brainstem function, and muscle strength (9). Each domain is scored from 0 to 3, yielding a total score range of 0–27, thereby providing a comprehensive evaluation of nonmotor symptoms.

Disease severity was stratified into three categories based on the peak mRS score (10): severe (mRS 4–5), moderate (mRS 2–3), and mild (mRS 0–1). Treatment response was classified as good, partial, or no response based on the composite improvement in mRS and CASE scores. The detailed criteria for severity classification and treatment response categorization are presented in Table 2.

**Table 2:** Classification of disease severity and treatment response criteria in AE.

Severity category	Definition
Severe neurological dysfunction	mRS score of 4 or 5 at the nadir of the disease.
Moderate neurological dysfunction	mRS score of 2 or 3 at the nadir of the disease.
Mild neurological dysfunction	mRS score of 1 at the nadir of the disease.

Treatment response assessment	Definition
A. Good response	Severe: mRS ≤3 with CASE score reduction.
	Moderate: mRS ≤2 with CASE score reduction.
	Mild: mRS maintained at 1 or 0 with CASE score reduction.
B. Partial response	Severe: mRS ≥4 despite CASE score reduction.
	Moderate: mRS ≥3 despite CASE score reduction.
C. No response	All severity levels: Unchanged or increased CASE score.

mRS, modified Rankin Scale; CASE, Clinical Assessment Scale in Autoimmune Encephalitis. Treatment response is assessed at discharge following first-line immunotherapy (intravenous methylprednisolone, intravenous immunoglobulin, plasma exchange, or a combination thereof).

### 2.5 Statistical analysis

All the statistical analyses were performed using SPSS version 26.0 (IBM Corp., Armonk, NY, United States). The Shapiro–Wilk test was used to assess the normality of the data distribution. Normally distributed continuous variables are presented as the mean ± standard deviation (SD) and were compared between groups using independent samples t tests. Nonnormally distributed continuous variables are expressed as medians with interquartile ranges (IQR) and were compared using the Mann–Whitney U test. Categorical variables are reported as frequencies and percentages [n (%)] and were analyzed using Pearson’s chi-square test or Fisher’s exact test, as appropriate. All the statistical tests were two-tailed, with a significance level set at *p* < 0.05.

## 3 Results

### 3.1 Clinical features

**Figure 1:**
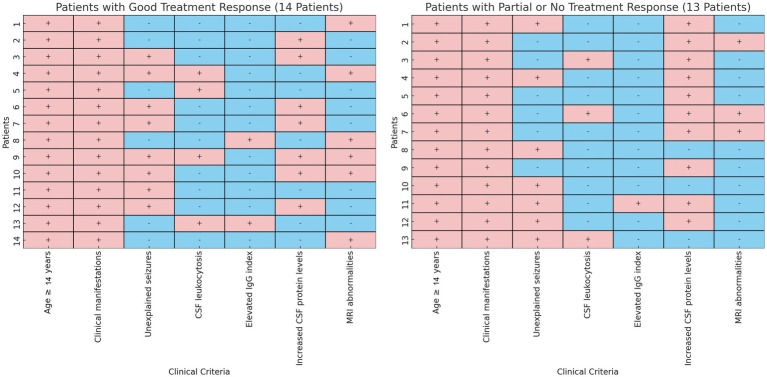
Analysis of inclusion criteria for treatment response in antibody-negative autoimmune encephalitis. This side-by-side heatmap comparison illustrates the clinical criteria assessment for two groups of antibody-negative autoimmune encephalitis patients: those with good treatment response (“Good Response,” *n* = 14) and those with suboptimal response (“Partial or No Response,” *n* = 13). Each panel delineates individual patients, indicating whether specific clinical criteria were met (soft red, +) or not met (soft blue, −).

Of the 60 patients with AE enrolled, 33 (55%) were antibody-positive and 27 (45%) were antibody-negative (Table 3). In the antibody-positive group, anti-NMDAR antibodies were most common (19/33, 57.6%), followed by anti-LGI1 (4/33, 12.1%), anti-CASPR2 and anti-GABAR (3/33 each, 9.1%), and anti-AMPAR1 (1/33, 3.0%). Two patients had multiple antibodies: one with NMDAR/GAD65 and another with CASPR2/NEUREXIN-3α antibodies. The analysis of inclusion criteria for treatment response in antibody-negative autoimmune encephalitis is detailed in Figure 1.

**Table 3:** Baseline characteristics and clinical features of patients with antibody negative and antibody positive patients.

Variables	Total (*n* = 60)	Antibody negative (*n* = 27)	Antibody positive (*n* = 33)	*p*-value
Age in years at onset [IQR]	34.00 (59.25–18.00)	34.00 (61.00–18.00)	34.00 (58.00–20.00)	0.920
Sex, male *n* (%)	32 (53.3)	18 (66.6)	14 (42.4)	0.061
Length of stay [IQR]	20.00 (33.25–14.00)	19.00 (29.00–14.00)	20.00 (34.00–14.00)	0.715
Onset to Immunotherapy [IQR]	13.00 (28.00–9.00)	10.50 (15.50–6.00)	14.00 (34.00–9.00)	0.152
Autoimmune diseases, *n* (%)	10 (16.7)	2 (7.4)	8 (24.2)	0.082
Active cancer, *n* (%)	4 (6.7)	2(7.4)	2(6.1)	0.835
Clinical profiles				
Initial CASE scores [IQR]	6.0 (9.00–4.00)	7.00 (11.00–4.00)	6.00 (8.00–3.00)	0.218
Peak CASE scores [IQR]	6.00 (10.25–4.00)	7.00 (12.50–5.00)	6.00 (9.00–4.00)	0.354
CASE at discharge [IQR]	3.50 (8.00–2.00)	4.00 (8.50–2.50)	3.00 (5.00–2.00)	0.132
Initial mRS scores, [IQR]	3.00 (4.00–2.00)	4.00 (4.00–2.00)	3.00 (4.00–2.00)	0.558
Peak mRS scores [IQR]	3.00 (5.00–2.00)	4.00 (5.00–2.00)	3.00 (5.00–2.00)	0.897
mRS at discharge [IQR]	2.00 (4.00–1.00)	2.00 (4.00–1.00)	2.00 (3.00–1.00)	0.489
Prodromal symptoms, *n* (%)	14 (23.3)	7 (25.9)	7 (21.2)	0.668
Onset of symptoms				0.761
Seizures, *n* (%)	18 (30.0)	9 (33.3)	9 (27.3)	
Psychiatric symptoms, *n* (%)	19 (30.0)	9 (33.3)	10 (27.3)	
Others symptoms, *n* (%)	23 (38.3)	9 (33.3)	14 (42.4)	
Symptom profiles				
Seizures, *n* (%)	32 (53.3)	18 (66.7)	14 (42.4)	0.061
Memory dysfunction, *n* (%)	12 (20.0)	3 (11.1)	9 (27.3)	0.119
Psychiatric symptoms, *n* (%)	33 (55.0)	14 (51.9)	19 (57.6)	0.795
Impaired consciousness, *n* (%)	14 (23.3)	6 (22.2)	8 (24.2)	0.854
Dyskinesia/dystonia, *n* (%)	17 (28.3)	9 (33.3)	8 (24.2)	0.437
Speech dysfunction, *n* (%)	5 (8.33)	1 (3.70)	4 (12.1)	0.367
Gait instability and ataxia, *n* (%)	8 (13.3)	4 (14.8)	4 (12.1)	>0.99
Cognitive discrimination, *n* (%)	29 (48.3)	12 (44.4)	17 (51.5)	0.586
Autonomic dysfunction, *n* (%)	11 (18.3)	2 (7.4)	9 (27.3)	0.100
Multiple symptoms (≥4)	17 (28.3)	4 (14.8)	13 (39.4)	***0.036***
MRI findings				
Any abnormality in MRI, *n* (%)	24 (40.0)	9 (33.3)	15 (45.5)	0.340
Limbic System, *n* (%)	16 (26.7)	5 (18.5)	11 (33.3)	0.197
Other Encephalitis, *n* (%)	11 (18.3)	6 (22.2)	5 (15.2)	0.712
EEG findings				
Pathological EEG overall, *n* (%)	48 (80.0)	21 (77.8)	27 (81.8)	0.697
Generalized slowing	30 (50.0)	12 (44.4)	18 (54.5)	0.436
Focal slowing, *n* (%)	5 (8.3)	4 (14.8)	1 (3.0)	0.241
Epileptic discharges, *n* (%)	13 (21.7)	5 (18.5)	8 (24.2)	0.592

Bold and italic values indicate statistical significance (*p* < 0.05). CASE, clinical assessment scale in autoimmune encephalitis; mRS, The modified rankin scale; EEG, Electroencephalogram; MRI, Magnetic resonance imaging.

The median age of the cohort was 34.0 years (IQR: 59.3–18.0), with 32 (53.3%) male patients. Age and sex distributions did not differ significantly between antibody-positive and antibody-negative groups. Autoimmune diseases were more prevalent in the antibody-positive group (24.2% vs. 7.4%, *p* = 0.082). Autoimmune thyroiditis was the most common comorbidity (4/60, 6.7%), followed by hyperthyroidism (3/60, 5.0%). Other autoimmune conditions included myasthenia gravis, Sjögren’s syndrome, and ankylosing spondylitis (1/60 each, 1.7%). Active malignancies were present in 4 patients (6.7%), with no significant difference between groups (6.1% vs. 7.4%, *p* = 0.835).

Prodromal symptoms occurred in 14 patients (23.3%), with a similar frequency in both groups (21.2% vs. 25.9%, *p* = 0.668). At disease onset, seizures and neuropsychiatric disturbances were the most common presenting symptoms (18/60 each, 30.0%). Throughout the disease course, neuropsychiatric disturbances (33/60, 55.0%) and seizures (32/60, 53.3%) remained the most prevalent symptoms, followed by cognitive impairment (29/60, 48.3%). Speech dysfunction was the least common manifestation (5/60, 8.3%). Seizures tended to occur more frequently in the antibody-negative group (66.7% vs. 42.4%, *p* = 0.061). The antibody-positive group showed significantly greater symptom diversity, with 13 (39.4%) patients presenting four or more symptoms compared to 4 (14.8%) in the antibody-negative group (*p* = 0.036).

EEG abnormalities were common in AE patients (48/60, 80%), while MRI abnormalities were less frequent (24/60, 40%). The frequency of EEG (81.8% vs. 77.8%, *p* = 0.690) and MRI (45.5% vs. 33.3%, *p* = 0.350) abnormalities did not differ significantly between antibody-positive and antibody-negative groups. EEG abnormalities primarily manifested as generalized slow waves (30/60, 50%), epileptiform discharges (13/60, 21.7%), and focal slow waves (5/60, 8.3%). MRI abnormalities predominantly involved the limbic system (16/60, 26.7%), particularly the medial temporal lobe structures. Inflammatory changes in other cerebral regions were observed in 11 patients (18.3%), affecting the frontal lobe, basal ganglia, thalamus, brainstem, and cerebellum.

### 3.2 Laboratory findings

Laboratory parameters between antibody-positive and antibody-negative AE patients are compared in Table 4. Serological analysis revealed no significant intergroup differences in complete blood count parameters or composite inflammatory markers (NLR and MLR). However, serum IgG concentrations were significantly higher in antibody-positive AE patients (median 11.63 vs. 10.26 g/L, *p* = 0.010).

**Table 4:** Comparison of laboratory parameters between antibody negative and antibody positive patients.

Variables	Total (*n* = 60)	Antibody negative (*n* = 27)	Antibody positive (*n* = 33)	*p*-value
Serum				
White Blood Cell, mean ± SD	8.44 ± 2.84	8.47 ± 3.25	8.42 ± 2.51	0.943
Neutrophils, [IQR]	5.62 (7.16–3.97)	5.36 (7.10–4.06)	5.93 (7.17–4.00)	0.710
Lymphocytes, mean ± SD	1.73 ± 0.63	1.75 ± 0.60	1.71 ± 0.66	0.827
Mononuclear, [IQR]	0.55 (0.71–0.45)	0.67 (0.72–0.51)	0.50 (0.60–0.44)	0.073
Neutrophil Ratio, mean ± SD	68.44 ± 11.04	67.19 ± 9.69	69.45 ± 12.08	0.435
C-reactive protein, [IQR]	1.40 (9.01–0.47)	3.28 (10.00–0.78)	1.03 (7.34–0.38)	0.093
Total Protein, mean ± SD	69.53 ± 7.11	68.72 ± 7.84	70.19 ± 6.50	0.432
Serum Albumin, mean ± SD	40.79 ± 4.64	40.84 ± 5.72	40.75 ± 3.62	0.943
Serum IgG, [IQR]	11.32 (12.73–10.02)	10.26 (12.08–9.25)	11.63 (13.16–10.78)	***0.010***
NLR, [IQR]	2.97 (4.93–2.16)	2.95 (4.82–2.20)	2.99 (5.88–2.13)	0.768
MLR, [IQR]	0.32 (0.50–0.23)	0.32 (0.52–0.25)	0.28 (0.46–0.20)	0.308
CSF				
CSF Total Protein, [IQR]	499.80 (660.98–315.27)	565.00 (752.90–394.00)	369.00 (582.80–263.30)	***0.025***
CSF Albumin, [IQR]	270.90 (412.77–147.35)	380.40 (478.00–234.55)	199.70(338.2–134.50)	***0.013***
CSF Lactate, [IQR]	1.76 (2.09–1.56)	1.88 (2.26–1.48)	1.75 (2.05–1.59)	0.563
CSF Chloride, [IQR]	125.35 (127.80–123.50)	125.10(127.25–123.60)	125.40(127.90–123.50)	0.466
CSF Glucose, [IQR]	3.82 (4.24–3.53)	3.82 (4.65–3.52)	3.82 (4.16–3.54)	0.761
CSF WBC, [IQR]	3.50 (24.75–2.00)	2.00 (8.00–1.00)	9.00 (52.00–3.00)	***0.014***
CSF IgG, [IQR]	40.75 (57.40–26.67)	44.25 (67.52–32.62,)	32.05 (51.88–21.48)	0.080
QAlb, [IQR]	6.30 (10.25–3.58)	8.72 (5.86, 11.68)	4.68 (3.50, 8.47)	***0.018***
IgG Index, [IQR]	0.51 (0.62–0.43)	0.50 (0.62–0.46)	0.51 (0.64–0.40)	0.848
IgG (loc), [IQR]	11.29 (14.04–10.07)	10.42 (13.54–9.65)	11.56 (14.51–10.84)	***0.017***
Increased CSF Pressure, *n* (%)	19 (31.7)	11 (40.7)	8 (24.2)	0.265
Increased CSF WBC, *n* (%)	26 (43.3)	7 (25.9)	19 (57.6)	***0.014***
Increased QAlb, *n* (%)	26 (43.3)	16 (59.3)	10 (30.3)	***0.024***
Increased IgG Index, *n* (%)	11 (18.3)	5 (18.5)	6 (18.2)	>0.99
Increased IgG SR, *n* (%)	14 (23.3)	9 (33.3)	5 (15.2)	0.129

Bold and italic values indicate statistical significance (*p* < 0.05). CSF, cerebrospinal fluid; WBC, white blood cell; IgG, immunoglobulin G; QAlb, albumin quotient; IgG SR, IgG synthesis rate; IgG (loc), IgG local synthesis. Reference ranges - Serum: WBC 3.5–9.5× 10^9/L, Neutrophils 1.8–6.3 × 10^9/L, Lymphocytes 1.1–3.2 × 10^9/L, Total Protein 65–85 g/L, Albumin 40–55 g/dL; CSF: WBC 0–8 cells/μL, Total protein 150–450 mg/L, Albumin 100–300 mg/L, Glucose 2.5–4.0 mmol/L, Chloride 120–130 mmol/L, Lactate 1.10–2.40 mmol/L, QAlb < 6.5 × 10^−3^ for < 40 years, <8 × 10^−3^ for 40–60 years, <9.3 × 10^−3^ for > 60 years, IgG-Index < 0.7, IgG (loc) 0–3.3 mg/24 h.

CSF analysis revealed marked differences between the groups. Antibody-positive AE patients had higher CSF leukocyte counts (median 9.00 vs. 2.00 cells/μL, *p* = 0.014) and local IgG synthesis rates (median 11.56 vs. 10.42, *p* = 0.017). Conversely, antibody-negative AE patients showed elevated CSF total protein (median 565.00 vs. 369.00 mg/L, *p* = 0.025) and albumin concentrations (median 380.40 vs. 199.70 mg/L, *p* = 0.013).

QAlb, an indicator of blood–brain barrier (BBB) integrity, was significantly higher in the antibody-negative AE group (median 8.72 vs. 4.68, *p* = 0.018). The proportion of patients with abnormal QAlb was also greater in this group (59.3% vs. 30.3%, *p* = 0.024), suggesting more severe BBB dysfunction in antibody-negative AE patients. CSF lactate, glucose, and chloride levels did not differ significantly between the groups.

### 3.3 Disease severity and treatment response

Treatment response and disease severity profiles are summarized in Table 5. The median time from symptom onset to immunotherapy initiation was 13.0 days (IQR: 28.0–9.0), with a median hospital stay of 20.0 days (IQR: 33.3–14.0). These temporal parameters did not differ significantly between antibody-positive and antibody-negative AE patients (*p* = 0.152 and *p* = 0.715, respectively).

**Table 5:** Analysis of treatment response and disease severity in patients.

Variables	Total (*n* = 60)	Antibody negative (*n* = 27)	Antibody positive (*n* = 33)	*p*-value
Treatment profiles				
Single first-line therapy, *n* (%)	22 (36.7)	15 (55.56)	7 (21.2)	***0.006***
Combined first-line therapy, *n* (%)	38(63.3)	13 (48.1)	24 (75.8)	***0.027***
Treatment modalities				
Immunoglobulin, *n* (%)	40 (66.67)	15 (55.56)	25 (75.76)	0.099
Steroids, *n* (%)	54 (90.00)	24 (88.89)	30 (90.91)	>0.99
Plasmapheresis, *n* (%)	7 (11.67)	1 (3.70)	6 (18.18)	0.182
Treatment response, *n* (%)				***0.022***
Good response, *n* (%)	35 (58.3)	14 (51,9)	21 (63.6)	
Partial response, *n* (%)	12 (20.0)	3 (11.1)	9 (27.3)	
No response, *n* (%)	13 (21.7)	10 (37.0)	3 (9.1)	
ΔCASE scores, [IQR]	2.00 (4.00–1.00)	1.00 (3.00–0.00)	2.00 (4.00–1.00)	***0.040***
ΔmRs scores, [IQR]	0.00 (1.25–0.00)	0.00 (1.00–0.00)	1.00 (2.00–0.00)	0.122
Severe disease (15/14)				
ΔCASE scores, [IQR]	3.00 (5.00–0.00)	1.00 (4.00–0.00)	4.50 (5.00–3.00)	***0.024***
ΔmRs scores, [IQR]	1.00 (2.00–0.00)	0.00 (2.00–0.00)	1.00 (2.00–0.25)	0.241
Moderate disease (6/13)				
ΔCASE scores, mean ± SD	2.11 ± 1.52	2.17 ± 1.47	2.23 ± 1.42	0.929
ΔmRs scores, [IQR]	1.00 (1.00–0.00)	0.50 (1.00–0.00)	1.00 (1.00–0.00)	0.480
Mild disease (6/6)				
ΔCASE scores, mean ± SD	1.00 (1.00–1.00)	1.00 (1.00–0.25)	1.00 (1.00–1.00)	0.390

Bold and italic values indicate statistical significance (*p* < 0.05). mRS, modified Rankin Scale; CASE, Clinical Assessment Scale in Autoimmune Encephalitis. Δ Mean Peak to Discharge Difference in CASE or mRS Scores. Severe disease: mRS score of 4 or 5 at the nadir of the disease. Moderate disease: mRS score of 2 or 3 at the nadir of the disease. Mild disease: mRS score of 1 at the nadir of the disease.

Treatment strategies varied significantly between groups. Antibody-positive AE patients more often received combination first-line therapy (75.8% vs. 48.1%, *p* = 0.027), while antibody-negative AE patients more frequently received monotherapy (55.6% vs. 21.2%, *p* = 0.006). Immunoglobulin therapy was more common in the antibody-positive group (75.8% vs. 55.6%, *p* = 0.099), whereas steroid use was similar (90.9% vs. 88.9%, *p* = 1.000). Plasma exchange was used more frequently in antibody-positive patients, the difference was not statistically significant (18.2% vs. 3.7%, *p* = 0.182).

Patients were classified as severe (29/60, 48.3%), moderate (19/60, 31.7%), or mild (12/60, 20.0%) based on their nadir mRS score. Although the disease severity distribution did not differ significantly between the groups (*p* = 0.362), the treatment response showed marked differences (*p* = 0.022). Antibody-positive patients more often exhibited good (63.6% vs. 51.9%) or partial (27.3% vs. 11.1%) responses, whereas antibody-negative patients more frequently showed no response (37.0% vs. 9.1%).

Although mRS score improvement did not differ significantly between groups, the improvement in CASE scores from nadir to discharge was greater in the antibody-positive group (median improvement: 2.00 vs. 1.00, *p* = 0.040). Subgroup analysis revealed that this difference was particularly pronounced in severely ill patients (median CASE score improvement: 4.50 vs. 1.00, *p* = 0.024) but not in moderately or mildly ill patients (*p* = 0.925 and *p* = 0.390, respectively).

## 4 Discussion

Our study identified significant differences in key clinical features between antibody-positive and antibody-negative AE patients, despite similar demographic characteristics, hospitalization duration, and neuroimaging findings. Antibody-positive AE patients showed a higher prevalence of concomitant autoimmune diseases, with Hashimoto’s thyroiditis (6.7%) and hyperthyroidism (5.0%) being the most common. This finding is consistent with previous research. Li et al. (6) reported a significantly higher incidence of immune-mediated disorders in antibody-positive AE patients compared to antibody-negative patients (*p* = 0.012). Similarly, Zhao et al. (11) found Hashimoto’s thyroiditis to be the most frequent coexisting autoimmune condition (5.42%) in a large-scale analysis of 517 antibody-positive AE patients.

These findings suggest a more extensive systemic immune dysregulation in antibody-positive AE patients. One plausible explanation is that these patients possess a specific autoimmune susceptibility profile. This susceptibility may not only increase the risk of autoimmune diseases in multiple organ systems but also promote the development of antibody-positive AE (12). This particular immune dysregulation appears to favor antibody-mediated autoimmune responses, targeting both neuronal surface or synaptic proteins and antigens in other organ systems. From a molecular perspective, this tendency might be attributed to specific alterations in B-cell function, resulting in enhanced production of various detectable autoantibodies (13).

Our findings demonstrated significant differences in symptom profiles and specific symptom incidence between antibody-positive and antibody-negative AE patients. Antibody-positive AE patients presented with more diverse clinical manifestations, with 39.4% exhibiting four or more symptoms, compared to 14.8% in the antibody-negative group. Conversely, antibody-negative AE patients had a higher incidence of seizures (66.7% vs. 42.4%). These observations are consistent with previous studies in Asian populations. Li et al. (6) reported a significantly higher seizure incidence in antibody-negative AE patients (81.25% vs. 54.55%), a finding corroborated by Guo et al. (14) (80.0% vs. 70.9%). Interestingly, these results contrast with data from European and North American cohorts. A multicenter study by Berger et al. (4) found a higher proportion of seizures in antibody-positive AE patients (54.1% vs. 36.1%), a trend supported by Probasco et al. (5). (50% vs. 31%). This geographical discrepancy suggests a potential influence of ethnic background on AE clinical manifestations, particularly seizure susceptibility in Asian patients with antibody-negative AE. However, this hypothesis requires validation through large-scale, multicenter studies to account for potential confounding factors and establish definitive ethnic-specific clinical patterns in AE.

Our study also identified significant differences in immune response patterns and BBB function between antibody-positive and antibody-negative AE patients. Serological analysis showed significantly elevated serum IgG concentrations in antibody-positive AE patients, despite similarities in most other indicators between the two groups. This finding is consistent with observations by Graus et al. (1). CSF analysis revealed further distinctions between the groups. Antibody-positive AE patients showed higher CSF leukocyte counts and elevated 24-h intrathecal IgG synthesis rates. The 24-h intrathecal IgG synthesis rate, a quantitative measure of intrathecal IgG production, excludes confounding effects of BBB damage and serum IgG levels, thus accurately reflecting endogenous IgG synthesis within the central nervous system (15). These elevated parameters suggest that antibody-positive AE patients experience both more pronounced peripheral immune activation and a more robust local immune response within the central nervous system.

Conversely, antibody-negative AE patients showed relatively low serum IgG levels and 24-h intrathecal IgG synthesis rates. This disparity may reflect different pathophysiological mechanisms underlying these AE subtypes. Mojžišová et al. (16) proposed that antibody-negative AE pathogenesis might involve non-antibody-mediated autoimmune processes, particularly adaptive cell-mediated responses. In this hypothesis, activated autoreactive T cells may recruit additional immune cells, triggering various effector pathways and inducing inflammatory neuronal damage without circulating antibodies. This theory could explain the comparable or lower serum IgG levels and intrathecal IgG synthesis rates observed in some antibody-negative AE patients compared to antibody-positive cases.

A notable finding of our study was the evidence of more severe BBB dysfunction in antibody-negative AE patients, corroborating previous research (4). The QAlb, a well-established indicator of BBB integrity, typically reflects increased BBB permeability when exceeding 7.00 (17, 18). Our analysis revealed significantly higher QAlb values in antibody-negative AE patients compared to their antibody-positive counterparts, with a greater proportion of antibody-negative patients exhibiting abnormal QAlb values. These observations strongly suggest more pronounced BBB dysfunction in antibody-negative AE. Elevated QAlb was accompanied by significant increases in both total protein and albumin concentrations in the CSF of antibody-negative AE patients. This phenomenon likely results directly from increased BBB permeability, allowing greater passage of peripheral blood proteins, including albumin and IgG, into the central nervous system (19).

Our analysis revealed significant differences in treatment strategies between antibody-positive and antibody-negative AE patients. Antibody-positive AE patients more frequently received combination first-line therapy compared to antibody-negative patients (75.8% vs. 48.1%, *p* = 0.027). Conversely, antibody-negative AE patients were more likely to receive monotherapy (55.6% vs. 21.2%, *p* = 0.006). Immunoglobulin therapy was administered more often in the antibody-positive group (75.8% vs. 55.6%, *p* = 0.099). These findings align with those reported by Berger et al. (4), who demonstrated a lower proportion of antibody-negative patients receiving first-line immunotherapy compared to antibody-positive cases (69.4% vs. 82.9%, *p* = 0.054). Berger et al. (4) also noted a significant disparity in the use of intravenous immunoglobulin (9.7% vs. 29.0%, *p* = 0.003). Several factors may contribute to the observed differences in treatment strategies between antibody-positive and antibody-negative AE patients. First, the absence of detectable antibodies in antibody-negative AE may introduce diagnostic uncertainty, potentially influencing clinical decision-making. Second, variations in healthcare systems across different countries and regions, combined with the high costs of immunoglobulin therapy and plasma exchange, may present economic barriers to more aggressive treatment approaches. These economic constraints may be particularly relevant for antibody-negative patients, where the lack of clear diagnostic biomarkers might further discourage the use of costly therapies.

Our findings demonstrate a higher proportion of treatment-responsive cases among antibody-positive AE patients, corroborating previous studies (4, 6). This disparity was particularly pronounced in severe cases, where improvements in CASE scores were significantly greater in the antibody-positive cohort. Conversely, approximately one-third of antibody-negative AE patients showed no response to immunotherapy. The mechanisms underlying the poorer prognosis in antibody-negative AE are likely multifactorial and remain incompletely understood. Treatment strategy differences may play a crucial role, as antibody-positive AE patients more frequently receive combination first-line therapy. Additionally, antibody-negative AE may encompass various pathological mechanisms, including low-titer antibodies, antibodies against unrecognized antigens, T-cell-mediated immune processes, and possibly non-immunogenic etiologies. This heterogeneity could explain the variable responses to standard immunotherapy protocols. Despite the overall lower treatment response rate in antibody-negative AE, approximately half of these patients benefit from immunotherapy. This observation emphasizes the importance of a comprehensive diagnostic approach, considering clinical manifestations and ancillary investigations, rather than relying solely on antibody test results. In cases of high clinical suspicion for AE, immunotherapy should be considered even in the absence of detectable antibodies, after rigorously excluding other potential diagnoses.

## 5 Conclusion

This study revealed distinct clinical and immunological profiles between antibody-positive and antibody-negative AE patients. Antibody-positive AE patients exhibited a more diverse symptom spectrum, elevated serum IgG concentrations, higher CSF leukocyte counts, and superior responses to immunotherapy. In contrast, antibody-negative AE patients demonstrated more severe blood–brain barrier dysfunction, evidenced by higher CSF total protein concentrations and albumin quotients. Importantly, our findings underscore that a negative antibody status should not preclude an AE diagnosis or immunotherapy consideration. In cases of high clinical suspicion for AE, after rigorous exclusion of other potential diagnoses, immunotherapy should be considered even in the absence of detectable antibodies.

## 6 Limitations

This study has several limitations. The single-center design and limited sample size may have reduced statistical power, potentially obscuring the significance of certain clinical indicators. While this study used both CASE and mRS to evaluate the immunotherapy response in AE patients, current assessment tools, particularly the mRS, may not fully capture the complex neurological and psychiatric manifestations of AE. Another consideration is the potential heterogeneity within the antibody-positive AE group. Clinical presentations of autoimmune encephalitis associated with different antibodies can vary significantly. For instance, AE associated with intracellular antigens often shows different responses to immunomodulating therapies compared to those associated with cell-surface antigens, potentially making some cases more similar to serum-negative AE. This heterogeneity within the antibody-positive group may have affected our comparisons. To address these limitations, future research should focus on multicenter, prospective studies with larger cohorts and randomized controlled trials, including more detailed subgroup analyses. The development and validation of AE-specific assessment tools that evaluate both neurological and psychiatric symptoms could improve the accuracy of disease severity assessment and treatment response evaluation. The development and application of more sensitive antibody detection techniques, coupled with mechanistic studies, could provide insights into AE heterogeneity and inform diagnostic and therapeutic strategies.

## Data Availability

The raw data supporting the conclusions of this article will be made available by the authors, without undue reservation.

## References

[1] Graus F Titulaer MJ Balu R Benseler S Bien CG Cellucci T. A clinical approach to diagnosis of autoimmune encephalitis. Lancet Neurol. (2016) 15:391–404. doi: 10.1016/S1474-4422(15)00401-9, PMID: 26906964 PMC5066574

[2] Dubey D Pittock SJ Kelly CR McKeon A Lopez-Chiriboga AS Lennon VA. Autoimmune encephalitis epidemiology and a comparison to infectious encephalitis. Ann Neurol. (2018) 83:166–77. doi: 10.1002/ana.25131, PMID: 29293273 PMC6011827

[3] Vora NM Holman RC Mehal JM Steiner CA Blanton J Sejvar J. Burden of encephalitis-associated hospitalizations in the United States, 1998-2010. Neurology. (2014) 82:443–51. doi: 10.1212/WNL.0000000000000086, PMID: 24384647

[4] Berger B Hauck S Runge K Tebartz van Elst L Rauer S Endres D. Therapy response in seronegative versus seropositive autoimmune encephalitis. Front Immunol. (2023) 14:1196110. doi: 10.3389/fimmu.2023.1196110, PMID: 37325671 PMC10264660

[5] Probasco JC Solnes L Nalluri A Cohen J Jones KM Zan E. Abnormal brain metabolism on FDG-PET/CT is a common early finding in autoimmune encephalitis. Neurol Neuroimmunol Neuroinflamm. (2017) 4:e352. doi: 10.1212/NXI.0000000000000352, PMID: 28567435 PMC5442608

[6] Li T Si Z Lu L Wang A. Autoimmune encephalitis: differences of clinical features between antibody-positive and antibody-negative conditions. Res. Sq. (2019). doi: 10.21203/rs.2.9140/v2

[7] Masciocchi S Businaro P Scaranzin S Morandi C Franciotta D Gastaldi M. General features, pathogenesis, and laboratory diagnostics of autoimmune encephalitis. Crit Rev Clin Lab Sci. (2024) 61:45–69. doi: 10.1080/10408363.2023.2247482, PMID: 37777038

[8] Seery N Butzkueven H O'Brien TJ Monif M. Rare antibody-mediated and seronegative autoimmune encephalitis: an update. Autoimmun Rev. (2022) 21:103118. doi: 10.1016/j.autrev.2022.103118, PMID: 35595048

[9] Lim JA Lee ST Moon J Jun JS Kim TJ Shin YW. Development of the clinical assessment scale in autoimmune encephalitis. Ann Neurol. (2019) 85:352–8. doi: 10.1002/ana.25421 30675918

[10] Popova E Mathai A Kannoth S Nair P Sasikumar S Gopinath S. Cerebrospinal fluid indices as predictors of treatment response in autoimmune encephalitis. Mult Scler Relat Disord. (2023) 79:104996. doi: 10.1016/j.msard.2023.104996, PMID: 37703639

[11] Zhao J Wang C Xu X Zhang Y Ren H Ren Z. Coexistence of autoimmune encephalitis and other systemic autoimmune diseases. Front Neurol. (2019) 10:1142. doi: 10.3389/fneur.2019.01142, PMID: 31736858 PMC6834766

[12] Somers EC Thomas SL Smeeth L Hall AJ. Autoimmune diseases co-occurring within individuals and within families: a systematic review. Epidemiology. (2006) 17:202–17. doi: 10.1097/01.ede.0000193605.93416.df, PMID: 16477262

[13] Arbuckle MR McClain MT Rubertone MV Scofield RH Dennis GJ James JA. Development of autoantibodies before the clinical onset of systemic lupus erythematosus. N Engl J Med. (2003) 349:1526–33. doi: 10.1056/NEJMoa021933 14561795

[14] Guo HL Wang GP. Comparison of clinical features between antibody-positive and antibody-negative autoimmune encephalitis patients. Anhui Med J. (2019) 40:1103–6. doi: 10.3969/j.issn.1000-0399.2019.10.007

[15] Conrad AJ Chiang EY Andeen LE Avolio C Walker SM Baumhefner RW. Quantitation of intrathecal measles virus IgG antibody synthesis rate: subacute sclerosing panencephalitis and multiple sclerosis. J Neuroimmunol. (1994) 54:99–108. doi: 10.1016/0165-5728(94)90236-4, PMID: 7929807

[16] Mojžišová H Krýsl D Hanzalová J Dargvainiene J Wandinger KP Leypoldt F. Antibody-negative autoimmune encephalitis: a single-center retrospective analysis. Neurol Neuroimmunol Neuroinflamm. (2023) 10:e200170. doi: 10.1212/NXI.0000000000200170, PMID: 37879962 PMC10605954

[17] Uher T Horakova D Tyblova M Zeman D Krasulova E Mrazova K. Increased albumin quotient (QAlb) in patients after first clinical event suggestive of multiple sclerosis is associated with development of brain atrophy and greater disability 48 months later. Mult Scler. (2016) 22:770–81. doi: 10.1177/1352458515601903, PMID: 26362893

[18] Link H Tibbling G. Principles of albumin and IgG analyses in neurological disorders. III. Evaluation of IgG synthesis within the central nervous system in multiple sclerosis. Scand J Clin Lab Invest. (1977) 37:397–401. doi: 10.1080/00365517709091498, PMID: 337461

[19] Gravely AA Cutting A Nugent S Grill J Carlson K Spoont M. Validity of PTSD diagnoses in VA administrative data: comparison of VA administrative PTSD diagnoses to self-reported PTSD checklist scores. J Rehabil Res Dev. (2011) 48:21–30. doi: 10.1682/jrrd.2009.08.0116, PMID: 21328160

